# Hepatic pleomorphic leiomyosarcoma after surgery for gastric gastrointestinal stromal tumor: a case report

**DOI:** 10.1186/s40792-019-0622-9

**Published:** 2019-04-16

**Authors:** Ryo Muranushi, Kouki Hoshino, Kei Hagiwara, Takahiro Yamanaka, Norihiro Ishii, Mariko Tsukagoshi, Takamichi Igarashi, Hiroshi Tanaka, Akira Watanabe, Norio Kubo, Kenichiro Araki, Norifumi Harimoto, Hayato Ikota, Kei Shibuya, Masaya Miyazaki, Ken Shirabe

**Affiliations:** 1 Department of General Surgical Science, Division of Hepatobiliary and Pancreatic Surgery, Graduate School of Medicine, Gunma University, 3-39-22 Showa-Machi, Maebashi, Gunma 371-8511 Japan; 2 Department of Human Pathology, Graduate School of Medicine, Gunma University, Maebashi, Japan; 3 Department of Diagnostic and interventional Radiology, Gunma University Hospital, Maebashi, Japan

**Keywords:** Pleomorphic hepatic leiomyosarcoma, Hepatectomy, Gastrointestinal stromal tumor

## Abstract

**Background:**

Pleomorphic leiomyosarcomas (PLMSs) are extremely rare tumors. We present the first case of hepatic primary PLMS after surgery for gastric gastrointestinal stromal tumor (GIST).

**Case presentation:**

The patient was a 62-year-old man who was referred to our hospital for resection of a hepatic tumor arising after gastric GIST surgery that was resistant to imatinib and sunitinib. A 40-mm tumor in the left lobe of the liver and three small nodules in the right lobe were detected. We performed hepatic left lobectomy and partial resections for three lesions. According to the histopathological and immunohistochemical findings and c-kit gene mutations analysis, the main tumor was diagnosed as a PLMS.

**Conclusion:**

It is necessary to consider the possibility that imatinib-resistant GIST recurrence lesions are a different kind of soft-tissue sarcoma. Accurate diagnosis is required to not miss the opportunity for radical excision of PLMS.

## Background

Pleomorphic leiomyosarcomas (PLMSs) account for 10% of leiomyosarcomas [1]. PLMSs often arise from skeletal muscle or retroperitoneal tissue in older people, have very poor prognosis, and are highly aggressiveness [1, 2]. Primary hepatic PLMSs are extremely rare. Although PLMSs are diagnosed by histopathological and immunohistochemical analyses, a differential diagnosis is difficult for patients with complicated backgrounds. Here, we present a case of hepatic resection for primary hepatic PLMS 2 years after surgery for gastric gastrointestinal stromal tumor (GIST).

## Case presentation

**Fig. 1:**
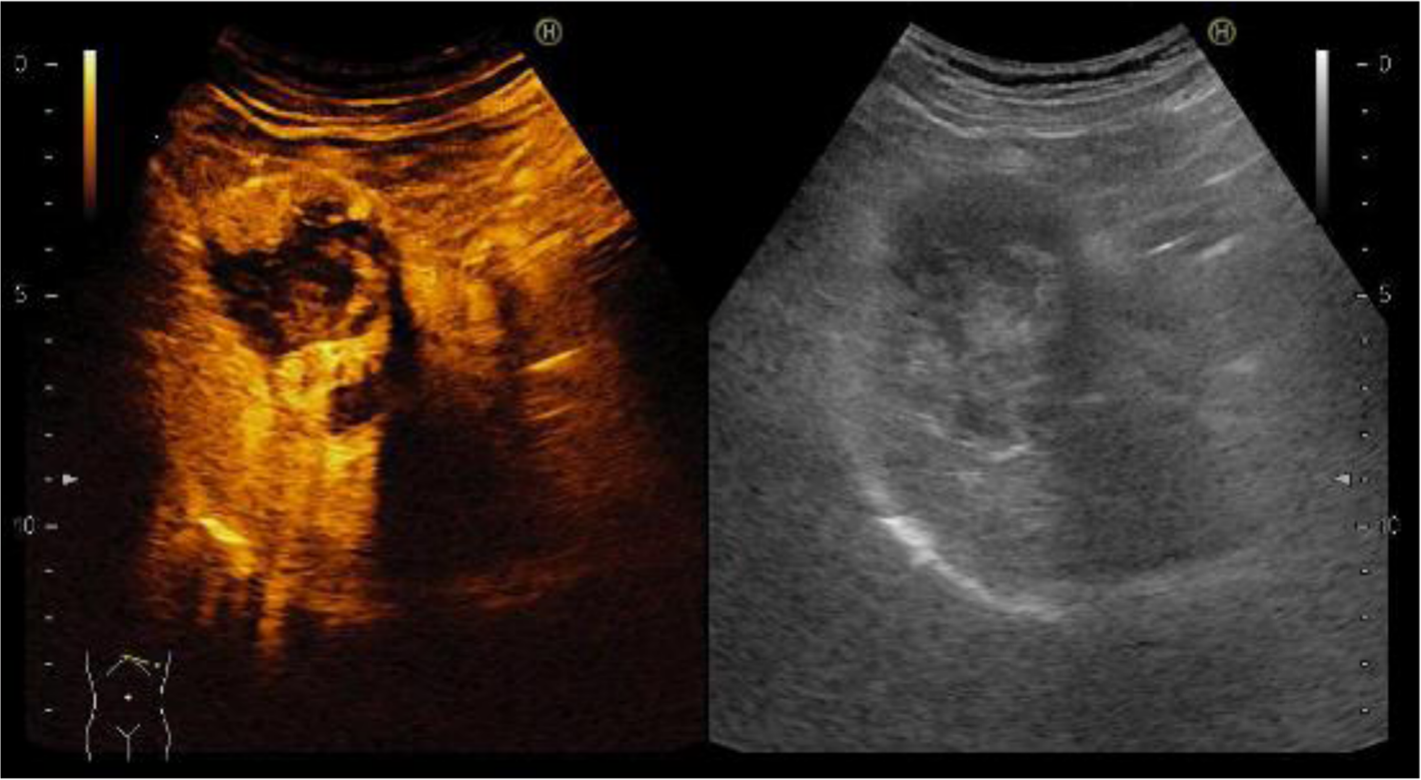
Abdominal ultrasonography findings. A 51-mm-wide tumor in hepatic segment 4 with heterogeneous echo was detected and it didn't present bloodstream increase. Sonazoid enhanced ultrasonography with hypervolemic contrasting pattern revealed that the tumor was enhanced in the early phase and washed out in the late phase

**Fig. 2:**
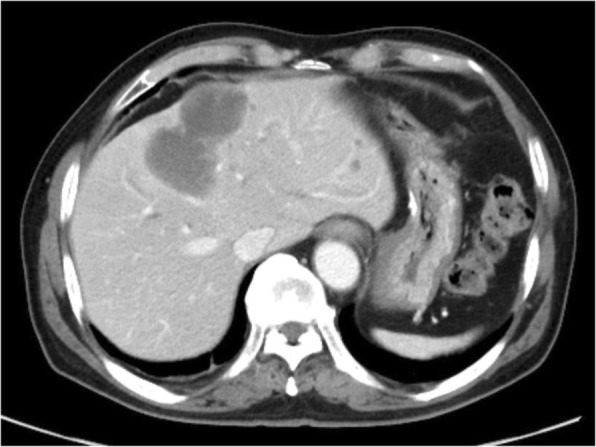
Enhanced computed tomography findings. A 40-mm-diameter tumor in hepatic segments 3 and 4 (S3+4) with enhanced solid nodule along the wall was detected. On the right side of the tumor, there was a 50-mm tumor, which suggested hemorrhagic cyst

**Fig. 3:**
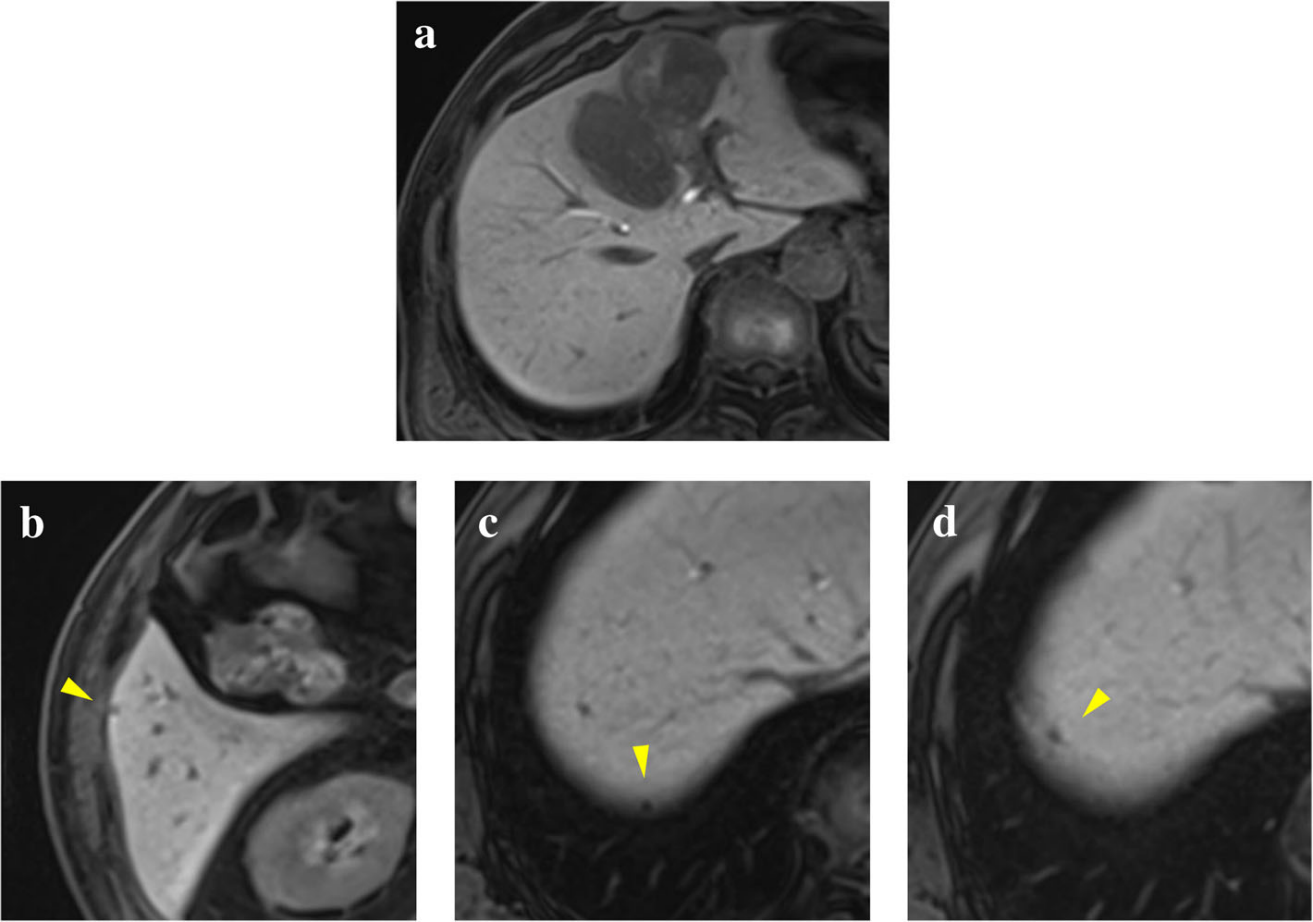
Gadolinium-enhanced magnetic resonance imaging findings. In addition to main tumor in S3+4 (**a**), small nodules that represented enhancement defect in the hepatocyte phase were detected in S6 (2**b**), S7 (2**c**), and S8 (2**d**)

**Fig. 4:**
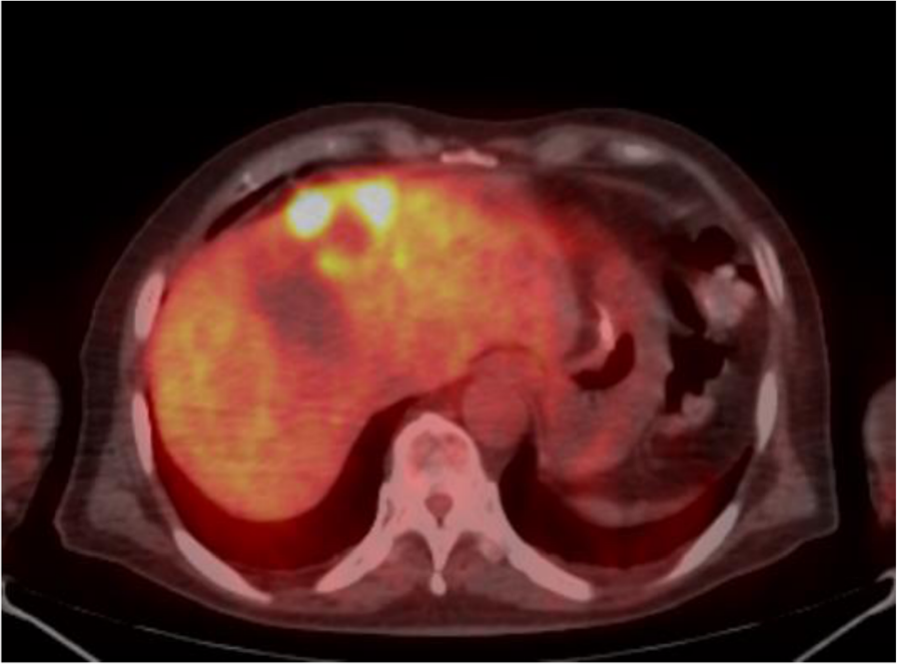
18F‑fluorodeoxyglucose positron-emission tomography findings. The S3+4 tumor verge represented high FDG accumulation (SUVmax=12.13)

**Fig. 5:**
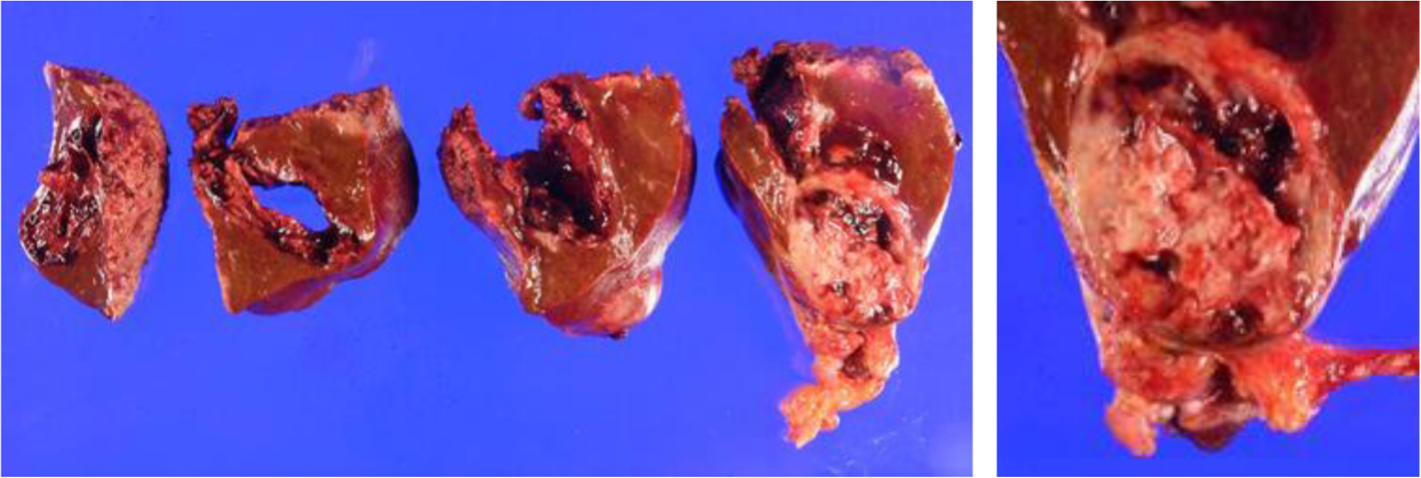
Photograph of resected specimen. The tumor measuring 73 mm × 65 mm × 36 mm in the resected left lobe showed a 40-mm white solid component and a 22-mm black nodule adjoining

**Fig. 6:**
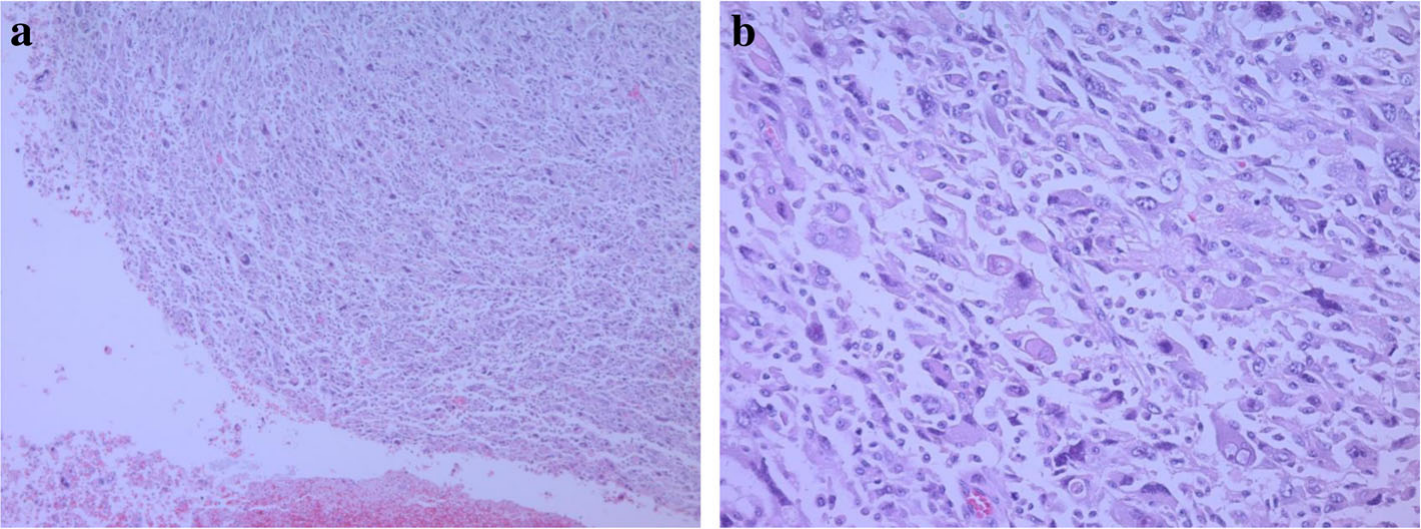
Histopathological findings. The spindle‑shaped cells with nuclear atypia and eosinophilic cytoplasm proliferated diffusely. The tumor cells were full of pleomorphism. **a**: Hematoxylin-Eosin staining, ×50 **b**: Hematoxylin-Eosin staining, ×200

**Fig. 7:**
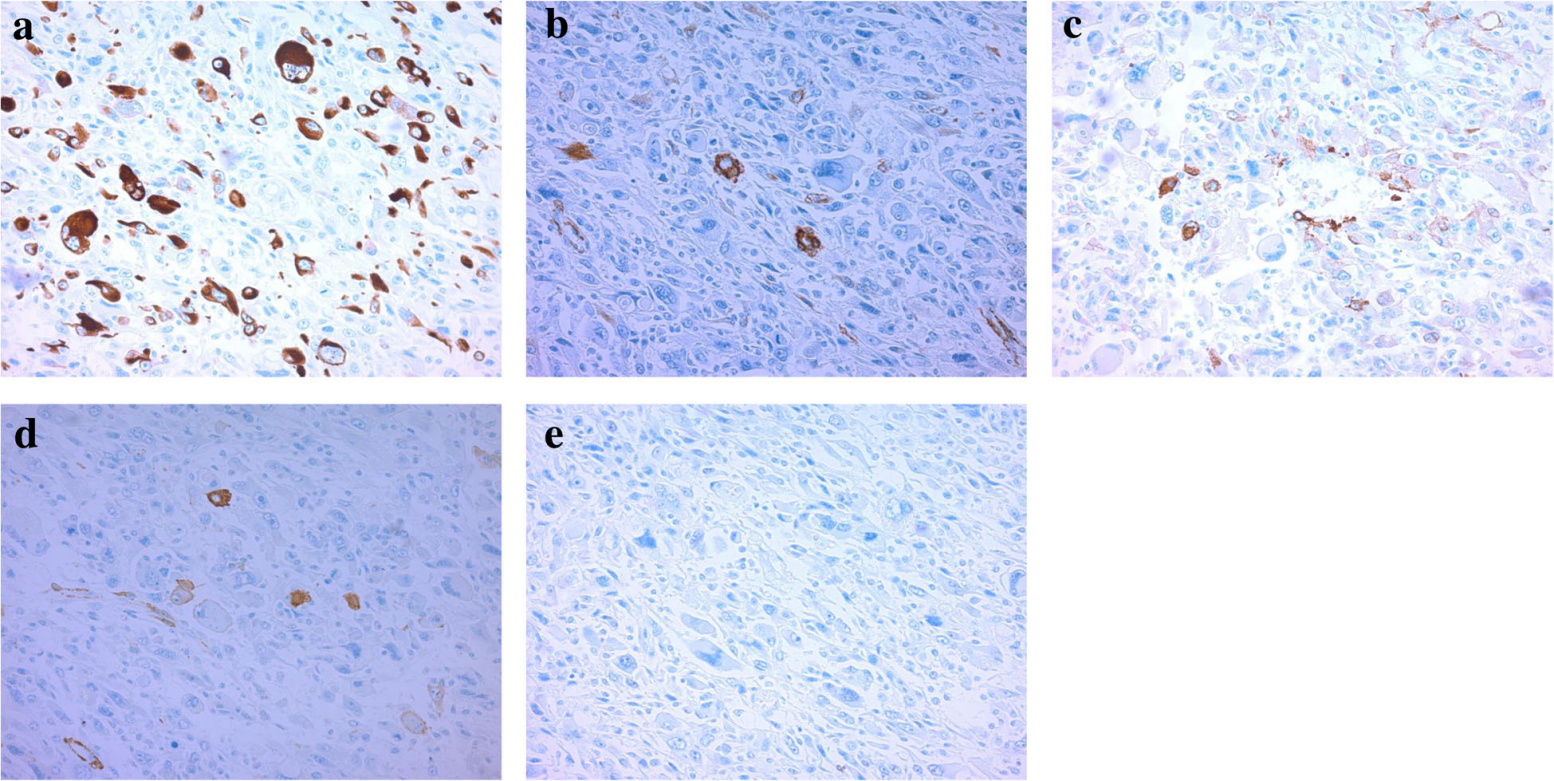
Immunohistochemical findings. Desmin was positive (**a**, ×200), α-smooth muscle actin (α-SMA) was slightly positive (**b**, ×200), and heavy-caldesmon (**c**, ×200) and muscle actin (HHF35) (**d**, ×200) was positive in 30% of the tumor tissue. Myogenic differentiation 1 (MyoD1) (**e**, ×200) did not present significant staining

The patient was a 62-year-old man who underwent gastric partial resection for GIST 2 years previously. Six months after the surgery, a single tumor emerged in the hepatic left lobe. Because it was thought that tumor was metastasis of the gastric GIST, he had started on imatinib based on the pathological and genetic evidence of the original lesion. Two months after beginning imatinib, the tumor had enlarged and the imatinib regimen was changed to sunitinib. Eleven months later, the tumor had grown further and he was referred to our hospital for surgery because the tumor was considered to be tolerant to tyrosine kinase inhibitors. His blood tests showed the following: aspartate aminotransferase, 32 U/L (normal range, 5 to 30 U/L); alanine phosphatase, 37 U/L (normal range, 10 to 30 U/L); total bilirubin 1.2 mg/dL, (normal range, 0.2 to 1.2 mg/dL); carcinoembryonic antigen, 3.5 ng/ml (normal range, < 5.0 ng/ml); and carbohydrate antigen 19–9, 8.0 U/ml (normal range, < 15 U/ml). An indocyanine green retention rate of 15 min was 15.1% with Child–Pugh grade A. Abdominal ultrasonography showed a 51-mm-wide tumor in hepatic segment 4 with heterogeneous echo and it didn't present bloodstreem increase. Sonazoid-enhanced ultrasonography with hypervolemic contrasting pattern revealed that the tumor was enhanced in the early phase and washed out in the late phase (Fig. 1). Enhanced computed tomography showed a 40-mm-diameter tumor in hepatic segments 3 and 4 (S3 + 4) with an enhanced solid nodule along the wall (Fig. 2). On the right side of the tumor, there was an additional 50-mm tumor, which suggested a hemorrhagic cyst (Fig. 2). Gadolinium-enhanced magnetic resonance imaging also showed an enhanced S3 + 4 tumor, a hemorrhagic cyst, and small nodules, which represented enhancement defects in the hepatocyte phase in hepatic segments 6 (S6), 7 (S7), and 8 (S8) (Fig. 3). ^18^F-Fluorodeoxyglucose positron-emission tomography showed a high FDG uptake lesion in only the S3 + 4 tumor verge. No evidence of metastasis from other organs was observed (Fig. 4). Although the imaging findings which suggested that the tumors were possibly other hepatic malignant tumors such as hepatocellular carcinoma had been considered, we diagnosed the tumors as hepatic metastases of gastric GIST because of the existence of multiple tumors and treatment progress. We performed hepatic left lobectomy and partial resections for three lesions. Intraoperative ultrasonography showed the tumor in hepatic medial segment and hematoma between the tumor and middle hepatic vein. The tumor measuring 73 mm × 65 mm × 36 mm in the resected left lobe showed a 40-mm white solid component, an adjoining 22-mm black nodule, and a cystic lesion with bleeding close to the margin (Fig. 5). The histopathological findings showed that the spindle-shaped cells with nuclear atypia and eosinophilic cytoplasm proliferated diffusely in the solid component (Fig. 6). Characteristically, the tumor cells were full of pleomorphism. The partially resected specimens did not contain tumor tissues. The S6 nodule was a cyst, while the S7 and S8 specimens represented dilated ducts. Accordingly, those lesions were not considered GIST metastases. Immunohistochemical analysis showed that desmin was positive, α-smooth muscle actin (α-SMA) was slightly positive, and heavy caldesmon and muscle actin (HHF35) were positive in 30% of the tumor tissue (Fig. 7). S-100 protein and myogenic differentiation 1 (MyoD1) did not present significant staining (Fig. 7), and c-kit and CD34 were negative.

In contrast with these findings, the gastric GIST specimen resected 2 years previously showed that although c-kit was positive and CD34 was weakly positive, the desmin, αSMA, and S-100 protein were negative. Additionally, c-kit gene mutations were not detected in the hepatic tumor tissue, whereas these mutations were positive in gastric GIST (Table 1).

**Table 1:** Immunohistochemical findings about PLMS and gastric GIST specimen resected 2 years previously. Heavy caldesmon, HHF35, and MyoD staining about GIST had not been performed

	PLMS	GIST
Desmin	Positive	Negative
α-SMA	Positive	Negative
Heavy caldesmon	Positive	
HHF35	Positive	
S-100	Negative	Negative
MyoD	Negative	
c-kit	Negative	Positive
CD34	Negative	Positive

According to these findings, we diagnosed the patient with primary hepatic PLMS. The postoperative course was good, and the patient was discharged 9 days after the operation. Because a chemotherapeutic strategy for PLMS has not been established, imatinib was administered as an adjuvant chemotherapy for GIST. Tumor recurrence was detected in the vicinity of the pancreatic head 10 months after the operation, and doxorubicin is now being administered.

## Discussion

Primary hepatic leiomyosarcomas account for 0.5 to 2% of all primary hepatic malignancies [3]. Nearly all primary sarcomas of the liver are angiosarcomas, epithelioid hemangioendotheliomas, and undifferentiated embryonal sarcomas or leiomyosarcomas, with more than 70% being angiosarcoma or undifferentiated embryonal sarcoma [4].

Leiomyosarcomas are malignant neoplasms that arise from smooth muscle. Hepatic leiomyosarcoma may arise from intrahepatic vascular structures, bile ducts, or ligamentum teres [5].

Histologically, PLMS contain a partial fascicular sequence of the spindle-shaped neoplastic cell typical of smooth muscle sarcoma, and most consist of polymorphic components similar to undifferentiated pleomorphic sarcoma. Immunohistochemical analysis is essential for diagnosis, is positive for SMA, desmin, and heavy caldesmon, and is negative for keratin and S-100 protein [1, 6].

PLMS treatment relies on surgery and aims to obtain negative tumor excision margins. It is important not to lose the opportunity for radical excision of PLMS. The surgical outcome for margin-negative resection (R0) extrapolated from two large series was 67% disease-specific survival at 5 years with 0% 3-year survival for patients who underwent R1 + resection [7, 8]. Successful initial surgical management is an important prognostic factor because complete surgical excision is associated with longer survival [9]. However, after surgical treatment, a high frequency of local recurrences is observed in PLMS (44 to 85%) [10, 11]. Neoadjuvant and adjuvant chemotherapy need to be established to improve the surgical rate after surgery.

Neoadjuvant chemotherapy is given to improve tumor resectability. The advantages are a reduction in the size of tumors and probability of a margin-negative resection [12]. Several randomized controlled trials (RCTs) exploring perioperative chemotherapy have been performed. Epirubicin and ifosfamide were effective in locally advanced high-risk primary sarcomas of the trunk and extremities that are high grade, > 5 cm, and deep in the tissue [13]. Additionally, the role of radiotherapy in patients with high-risk soft tissue sarcomas of the extremities and trunk is supported by findings from several RCTs [13].

Adjuvant systemic therapies have been tested to reduce the risk of metastatic spread after surgery with or without radiotherapy in several RCTs. Recent trials have tested anthracycline combined with ifosfamide; these treatment strategies offer a survival benefit of 5 to 10% to patients, which was considered unsatisfactory, particularly when balanced against high-grade toxicity [14].

Only approximately 70 cases of primary hepatic leiomyosarcomas are reported in the English literature [15]. To our knowledge, this is the first reported case of primary hepatic PLMS. Additionally, this is a valuable case in which PLMS emerged after surgery for GIST. Although it was considered that these had occurred successively, that made differential diagnosis difficult. Accurate diagnosis is required because of the significant difference in the postoperative prognosis and therapies between the two diseases. The present case shows that immunohistochemical and c-kit gene mutation analysis are effective in differential diagnosis. The first-line treatment choice for the recurrence of GIST is surgery for resectable lesions. If the tumors showed tolerance to imatinib in unoperated cases, they should be examined using pathological analysis as soon as possible considering the possibility of different soft-tissue sarcomas merging.

## Conclusion

We performed hepatectomy for primary hepatic PLMS after surgery for gastric GIST. It is necessary to consider that recurring imatinib-resistant GIST lesions may be different tumors.

## Abbreviations

GIST: Gastrointestinal stromal tumor
PLMS: Pleomorphic leiomyosarcomas
RCTs: Randomized controlled trials
α-SMA: α-smooth muscle actin
